# Spatiotemporal expression patterns of genes coding for plasmalemmal chloride transporters and channels in neurological diseases

**DOI:** 10.1186/s13041-023-01018-w

**Published:** 2023-03-18

**Authors:** Yanruo Huang, Qihang Wang, Yunsong Peng, Wenjie Du, Qi Wang, Jiangtao Qi, Zijian Hao, Yingwei Wang

**Affiliations:** 1 Department of Anesthesiology, Huashan Hospital, Fudan University, Shanghai, 200040 China; 2 State Key Laboratory of Molecular Biology, Shanghai Institute of Biochemistry and Cell Biology, Center for Excellence in Molecular Cell Science, Chinese Academy of Sciences, Shanghai, 200031 China; 3 University of Chinese Academy of Sciences, Beijing, 100049 China; 4 Division of Life Sciences and Medicine, School of Biomedical Engineering (Suzhou), University of Science and Technology of China, Hefei, 230026 China; 5 Medical Imaging Department, Suzhou Institute of Biomedical Engineering and Technology, Chinese Academy of Sciences, Suzhou, 215163 China; 6 Institute of Science and Technology for Brain-Inspired Intelligence, Fudan University, Shanghai, 200433 China; 7 MOE Frontiers Center for Brain Science, Fudan University, Shanghai, 200433 China

**Keywords:** Neurodevelopment, Gene expression pattern, Chloride transporter, Chloride channel, SLC12A2

## Abstract

Neuronal voltage changes which are dependent on chloride transporters and channels are involved in forming neural functions during early development and maintaining their stability until adulthood. The intracellular chloride concentration maintains a steady state, which is delicately regulated by various genes coding for chloride transporters and channels (GClTC) on the plasmalemma; however, the synergistic effect of these genes in central nervous system disorders remains unclear. In this study, we first defined 10 gene clusters with similar temporal expression patterns, and identified 41 GClTC related to brain developmental process. Then, we found 4 clusters containing 22 GClTC were enriched for the neuronal functions. The GClTC from different clusters presented distinct cell type preferences and anatomical heterogeneity. We also observed strong correlations between clustered genes and diseases, most of which were nervous system disorders. Finally, we found that one of the most well-known GClTC, *SLC12A2*, had a more profound effect on glial cell-related diseases than on neuron-related diseases, which was in accordance with our observation that *SLC12A2* was mainly expressed in oligodendrocytes during brain development. Our findings provide a more comprehensive understanding of the temporal and spatial expression characteristics of GClTC, which can help us understand the complex roles of GClTC in the development of the healthy human brain and the etiology of brain disorders.

**Supplementary Information:**

The online version contains supplementary material available at 10.1186/s13041-023-01018-w.

## Introduction

Chloride, the most abundant physiological anion, is involved in various biophysical processes, including osmotic equilibrium, membrane potential modulation, and intracellular pH maintenance [1]. The electrochemical gradient of chloride is determined by the extracellular and intracellular concentrations as dictated by the Nernst equation, and the corresponding equilibrium potential (ECl) and the membrane potential (Vm) set the driving force (Vm-ECl) of Cl^−^ currents mediated by γ-aminobutyric acid sub-type A and glycine receptors (GABA_A_Rs and GlyRs) [2].

During brain development, the intracellular chloride concentration of neural cells changes dynamically. This dynamic variance plays a pivotal role in the regulation of neural function [3, 4]. For example, the action of γ-aminobutyric acid (GABA) critically depends on the changes of intracellular chloride concentration. In the early stages of development, the intracellular chloride concentration is relatively high. ECl is above Vm, and thus GABA_A_R activation results in chloride ions efflux and neuronal depolarization. Depolarizing response not only promotes developmental processes, but also governs developmental switching points, from proliferation to migration, from migration to differentiation, and finally from differentiation to synapse formation [5]. We in the later stage of development, the level of intracellular chloride is relatively low. ECl is lower than Vm, which allows adult neurons to hyperpolarize in response to GABA_A_R activation by GABA. Hyperpolarizing response regulates the electrical activity and tightens neuronal plasticity to optimize neuronal networks [6, 7].

Although multiple factors can influence chloride regulation [8], the chloride channels and transporters are the direct executors to mediate chloride ions to enter or exit the cells to maintain intracellular chloride homeostasis, such as ligand-gated channels, the cystic fibrosis transmembrane conductance regulator (CFTR), and cation-chloride co-transporters [9, 10]. In this study, we use the abbreviation “GClTC” to refer to the genes coding for plasmalemmal chloride transporters and channels. Several studies have highlighted that the dysfunction of these GClTC leads to central nervous system (CNS) diseases, including autism spectrum disorder (ASD), neonatal epilepsy, and schizophrenia [11–14]. However, the underlying mechanisms are not fully understood.

Chloride homeostasis is affected by the transcriptional regulations of multiple GClTC. However, current studies have focused more on the roles of single genes in a restricted time and space, instead of the roles of a group of GClTC on a dynamic spatio-temporal scale [15]. In this study, we first clustered the genes with similar temporal expression patterns and focused on 4 out of 10 clusters whose functions were related to the CNS. Then we investigated the biological roles of GClTC in those clusters via Gene Ontology (GO) database, which provides a specific biological information of protein functions. Finally, based on the cellular and spatial distribution characteristics of GClTC, we expounded on the relationship between the gene expression features and CNS diseases, which provided a more comprehensive view of the underlying mechanisms of CNS disorders induced by disrupted chloride homeostasis.

## Methods

### Gene expression profile acquisition

**Fig. 1:**
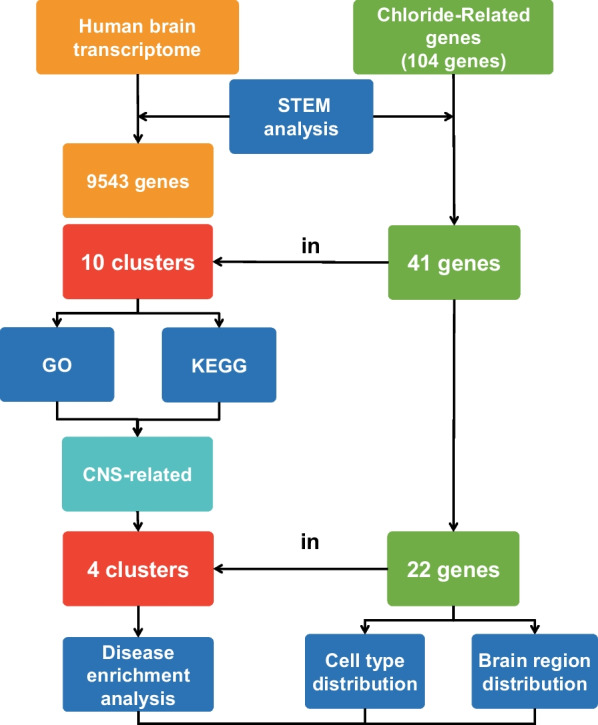
A multi-stage analysis methodology was employed in this study. Transcriptomic datasets of the developing human brain were collected from the Atlas of the Developing Human Brain. The GClTC were defined using the Gene Ontology database. STEM software was used to cluster genes with similar temporal expression patterns. Functional enrichment analyses of different clusters were performed to identify significantly-enriched pathways and GO terms. We selected the clusters related to the CNS and explored the cell type and brain region distribution of GClTC in those clusters. Combining those expression features with enriched diseases, we illustrate their intrinsic connections. *GO* Gene Ontology, *KEGG* Kyoto encyclopedia of genes and genomes, *CNS* Central nervous system, *STEM* short time-series expression miner

To explore the specific gene expression in the human brain at different developmental periods, RNA-seq data from different developmental periods were downloaded from the Atlas of the Developing Human Brain [16] (http://www.brainspan.org/static/download). Brain single-cell transcriptome data were obtained from the Multiple Cortical Areas-Smart-Seq [17] (https://portal.brain-map.org/atlases-and-data/rnaseq). The gene expression landscape, cell type heterogeneity, and temporal dynamics across the human brain were obtained using the Spatio-Temporal cell Atlas of the human Brain (STAB) [18]. Heatmaps of the gene expression profiles and enrichment analyses were generated using TBtools [19]. The workflow chart is shown in Fig. 1.

### Gene expression pattern analysis

Gene expression patterns during development were analyzed using Short Time-Series Expression Miner (STEM) software [20]. The options were set to default because STEM already provided optimal results for both biological and simulated data. The expression parameter used in this analysis was the reads per kilobase million (RPKM) value, and a *p*-value < 0.05 for the clustered profile was considered significant. The related R packages were used for data visualization.

### Functions, pathways, and diseases associated with developmental gene expression patterns

Gene Ontology (GO) enrichment analysis and Kyoto Encyclopedia of Genes and Genomes (KEGG) pathway enrichment analysis of different clusters were performed using the R package “clusterprofiler”, and *p*-values for the representative GO and KEGG terms shown in this study were adjusted for multiple testing with the appropriate Benjamini–Hochberg correction. The functional categories and pathways with adjusted *p*-values < 0.05 were deemed to be significantly enriched. Gene-Disease Association (GDA) analysis of differentially expressed genes was performed according to the DisGeNET association-type ontology [21]. In this analysis, a *q*-value < 0.05 was considered significant.

### Spatial gene expression analysis

The spatial expression information of genes in the brain was obtained using the Allen Institute Human Brain Atlas [22]. Whole-brain gene expression maps for GClTC were downloaded from the Neurosynth [23] (https://neurosynth.org/genes/). Functional connectivity was offered by Neurosynth, and we adopted this index to determine the relative strengths of the associations. This analysis yielded the Pearson correlation coefficient (*r*) between the whole-brain (uncorrected) reverse-inference map for each term and the functional connectivity map seeded at the current location. Gene maps and whole-brain maps were merged using Python.

### Gene expression analysis in specific diseases

Specific disease gene expression data were acquired from the Gene Expression Omnibus (GEO) database GSE108000 [24] and GSE122228 [25]. Gene set enrichment analysis (GSEA) was conducted to identify differences in the enrichment of GClTC between the control and disease groups. Shapiro–Wilk test was applied to assess data normality. Bartlett's test was applied to estimate the variance homogeneity. The Student’s t-test or Wilcoxon test was used to analyze the statistical difference. These statistical analyses were performed by R package "stats". Differences were considered statistically significant if *p* < 0.05.

## Results

### A developmental gene expression landscape of brain

Similar neural functions are often generated in a specific time window, and associative transcriptomic states present consistent neuronal development dynamics [26, 27]. Studying transcriptional regulation across brain development periods can facilitate the understanding of brain functions and cellular mechanisms that underlie brain diseases [28]. Short time-series expression miner (STEM) software is widely used to study gene expression data of biological processes with short time series [20]. It adopts a clustering algorithm to classify time series gene expression trends. In this study, we used STEM software to cluster genes with similar temporal expression patterns, whose expression changes synchronously during brain development.

**Fig. 2:**
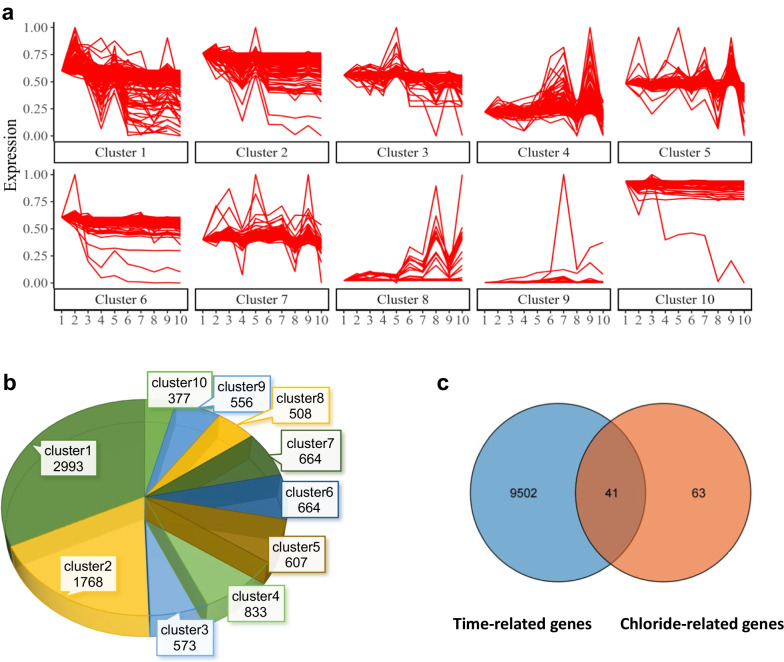
Temporal gene expression profiles in the developing brain. **a** The series of diagrams illustrates the developmental changes in gene expression patterns during the developmental period. The x-axis represents 10 developmental periods. The numbers 1 to 10 correspond to12 pcw, 13 pcw, 17 pcw, 21 pcw, 24 pcw, 4 mos, 1 yrs, 3 yrs, 8 yrs, 18 yrs, respectively. The y-axis represents the normalized gene expression changes at each period. **b** Pie chart showing the number of genes in each cluster in (**a**). **c** Venn diagram showing the temporal expression genes (9543 genes) intersected with GClTC (104 genes). *pcw* post-conception week, *mos* months, *yrs* years

The transcriptomic data for human brain genes were obtained from the Atlas of the Developing Human Brain database. We focused on transcriptomic changes during hippocampal development as it is a representative biological process in the development of brain regions [29]. Ten time points (from 12 post-conception weeks to 18 years) representing different developmental periods were selected for further analysis (Additional file 1: Table S1). A total of 9543 genes were screened out by STEM, and we defined these genes with temporal expression features as “developmental-stage dependent genes”. They were further classified into different groups as clusters, according to their unique time sequence change characteristics and different expression kinetics. 10 clusters with statistical differences were selected. They were ordered by their statistical significance in Fig. 2a and Additional file 2: Table S2. The number of clusters is marked in a pie chart (Fig. 2b).

Considering the intracellular chloride ion concentration is directly mediated by chloride transporters and channels, we focus on their GClTC in our study and explore their roles during development. We first identified the target genes using the term “chloride transmembrane transport (GO:1902476): the process in which chloride is transported across a membrane” in the Gene Ontology database [30]. 454 genes of “Homo sapiens” were included in this term. After ruling out the duplicated gene names, 104 GClTC were finally selected for further analysis (Additional file 3: Table S3). Next, we intersected these 104 GClTC with 9543 developmental-stage dependent genes. A total of 41 GClTC were identified (Fig. 2c and Table 1). They were evenly distributed among different clusters, and no obvious clustering was observed in one group.

**Table 1:** Classification of GClTC

Cluster1	Cluster2	Cluster3	Cluster4	Cluster5	Cluster6	Cluster7	Cluster8	Cluster9	Cluster10
*SLC26A10*	*TTYH1*	*GABRG3*	*ANO3*	*GABRG2*	*LRRC8A*	*SLC1A1*	*TTYH2*	*GABRB2*	*TTYH3*
*SLC12A9*	*CLIC1*	*GABRA3*	*GABRB1*	*GABRG1*	*SLC12A6*	*CLCN6*	*FXYD1*	*GLRA3*	
*SLC26A6*		*SLC1A4*	*GABRA2*	*ANO5*	*SLC12A7*		*APOL1*	*GABRA1*	
*ANO8*		*CLCN7*	*GLRB*				*CLCA4*	*GABRA5*	
*GLRA2*			*SLC4A8*				*SLC12A2*	*GABRA4*	
*ANO10*								*SLC12A5*	
								*CLIC6*	
								*GABRD*	
								*GABRQ*	

*SLC* Solute Carrier Family, *ANO* Anoctamin, *GLR* Glycine Receptor, *TTYH* Tweety Family Member, *CLIC* Chloride Intracellular Channel, *GABR* Gamma-Aminobutyric Acid Type A Receptor, *LRRC8A* Leucine Rich Repeat Containing 8 Family Member A, *FXYD* FXYD Domain Containing Ion Transport Regulator, *APOL* Apolipoprotein.

### Pathways associated with expression patterns

Genes with temporal expression features may be involved in time-dependent developmental functions. Therefore, we conducted GO and KEGG enrichment analyses of the genes from 10 clusters. Each cluster exhibited unique biological characteristics (Additional file 8: Fig. S1 and Additional file 4: Table S4).

**Fig. 3:**
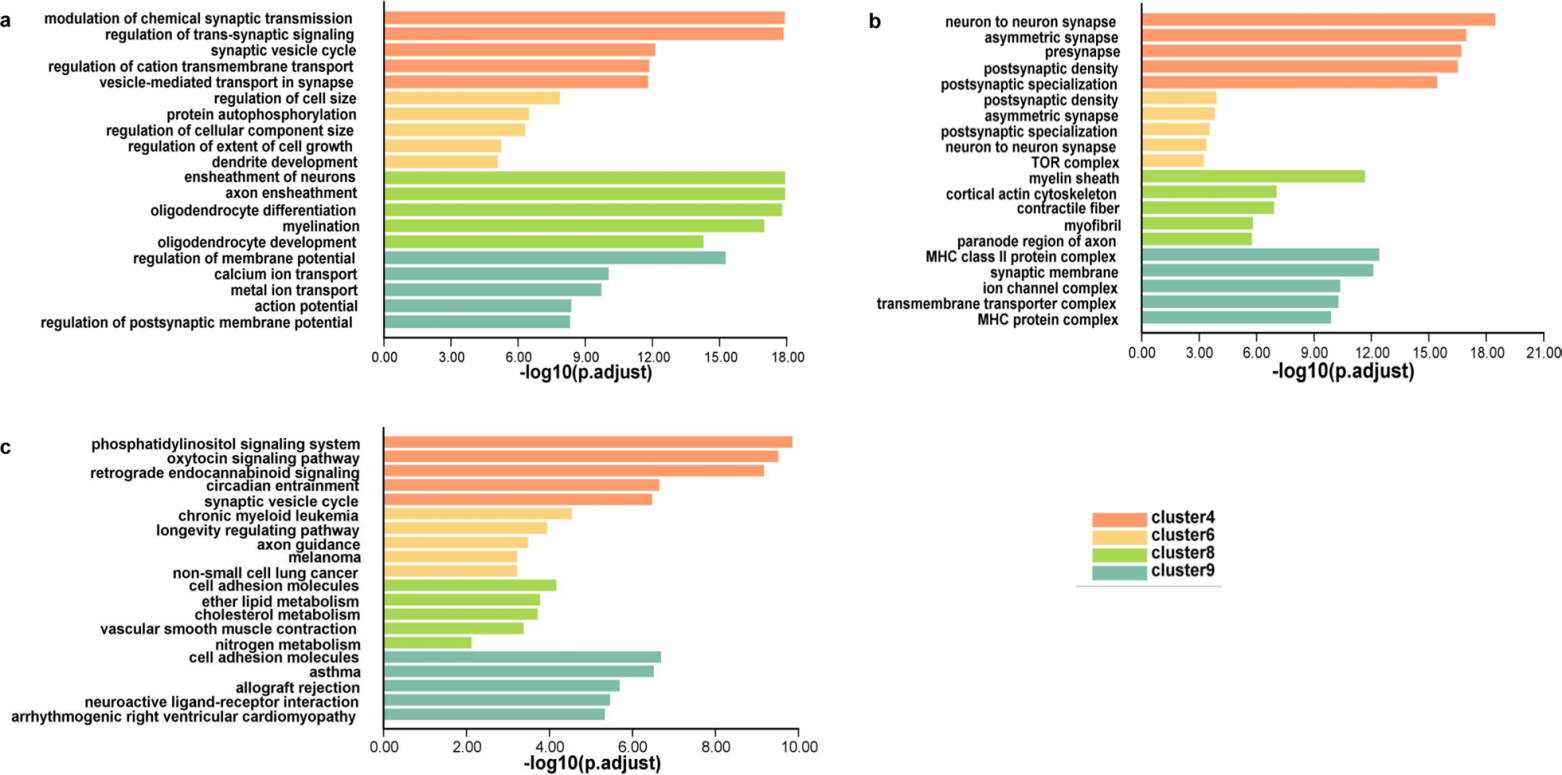
Molecular characteristics of the developmental expression patterns. **a** GO analysis (biological processes, BP) of genes in clusters 4, 6, 8, and 9. **b** GO analysis (cellular components, CC) of the genes in clusters 4, 6, 8, and 9. **c** KEGG analysis of the genes in clusters 4, 6, 8, and 9

Notably, we found genes from clusters 4, 6, 8, and 9 were mainly enriched for CNS-specific terms (Fig. 3). For example, in biological processes (BP) most terms of clusters 4 and 9 were related to signal regulation in the CNS. Cluster 6 contained terms for the regulation of cell morphogenesis. Cluster 8 included terms related to ensheathment formation, which belonged to non-neuronal cell components. Moreover, the enriched cellular component (CC) analysis corresponded with the BP-associated locations, such as synapse in clusters 4 and 9, and myelin sheath in cluster 8. KEGG analysis revealed that clusters 4, 6, and 9 tended to trigger pathways involved in regulating neural signals, such as phosphatidylinositol signaling pathway, oxytocin signaling pathway, and axon guidance. According to the above results, the genes in clusters 4, 6, 8, and 9 may be of great importance in neural functions. We observed that 22 GClTC were present in those 4 clusters, suggesting they may regulate the corresponding neuronal functions.

### Heterogeneity of GClTC expression across cell subtypes

**Fig. 4:**
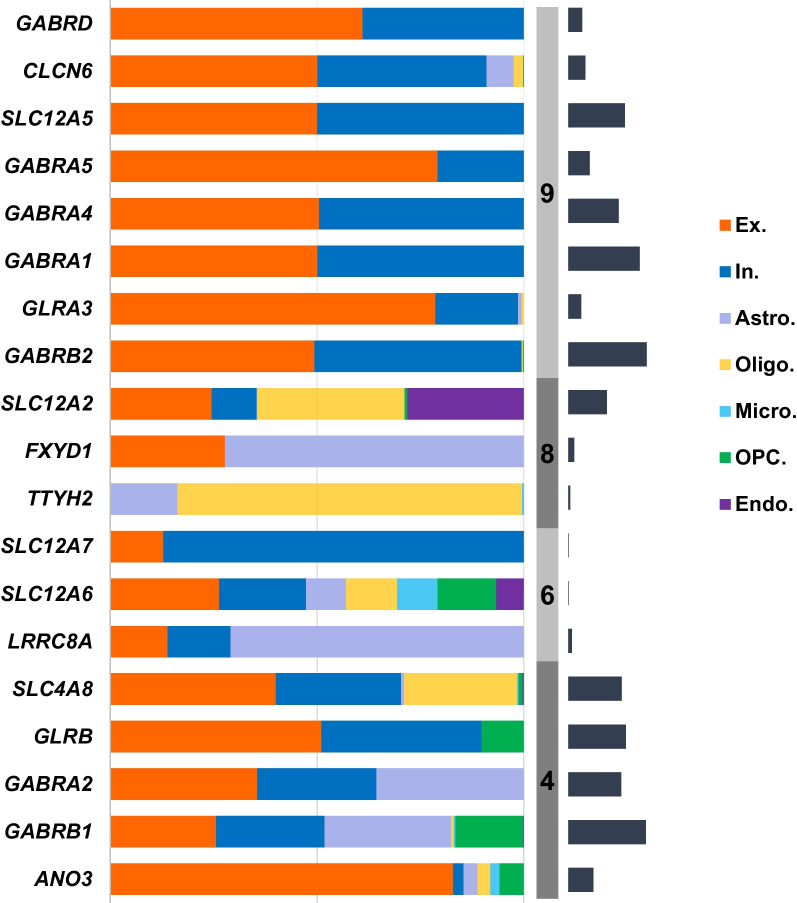
Expression levels of GClTC in different cell types. The salmon-colored bars indicate the percentage of gene expression in each cell type. The gray-colored bars indicate the clusters. The dark blue-colored bars on the right indicate the relative expression of gene levels in the brain. Genes with extremely low expression were removed. *Ex* excitatory neuron, *In* inhibitory neuron, *Astro* astrocyte, *Oligo* oligodendrocyte, *Micro* microglial cell, *OPC* oligodendrocyte precursor cell, *Endo* endotheliocyte

We then investigated the distribution of GClTC involved in the regulation of CNS functions in different cell types. The single-cell transcriptomics data were obtained from the Multiple Cortical Areas-Smart-Seq [17]. As shown in Fig. 4, the genes in clusters 4 and 6 showed no preference for certain cell types; however, the genes in cluster 9 were expressed mainly in neurons, with minimal expression in non-neuronal cells. The genes in cluster 8 were mainly expressed in glial cells. To investigate whether these genes and the specific cell types were determined during early development, we selected some GClTC using the Spatio-Temporal cell Atlas of the human Brain (STAB) to evaluate their expression dynamics in different cell types. Representative genes selected from the different clusters are shown in Additional file 9: Fig. S2. The results indicated that remarkable changes in expression levels during development were detected in specific cell types, consistent with the distribution of these genes in adults.

### Spatial specificity of GClTC

**Fig. 5:**
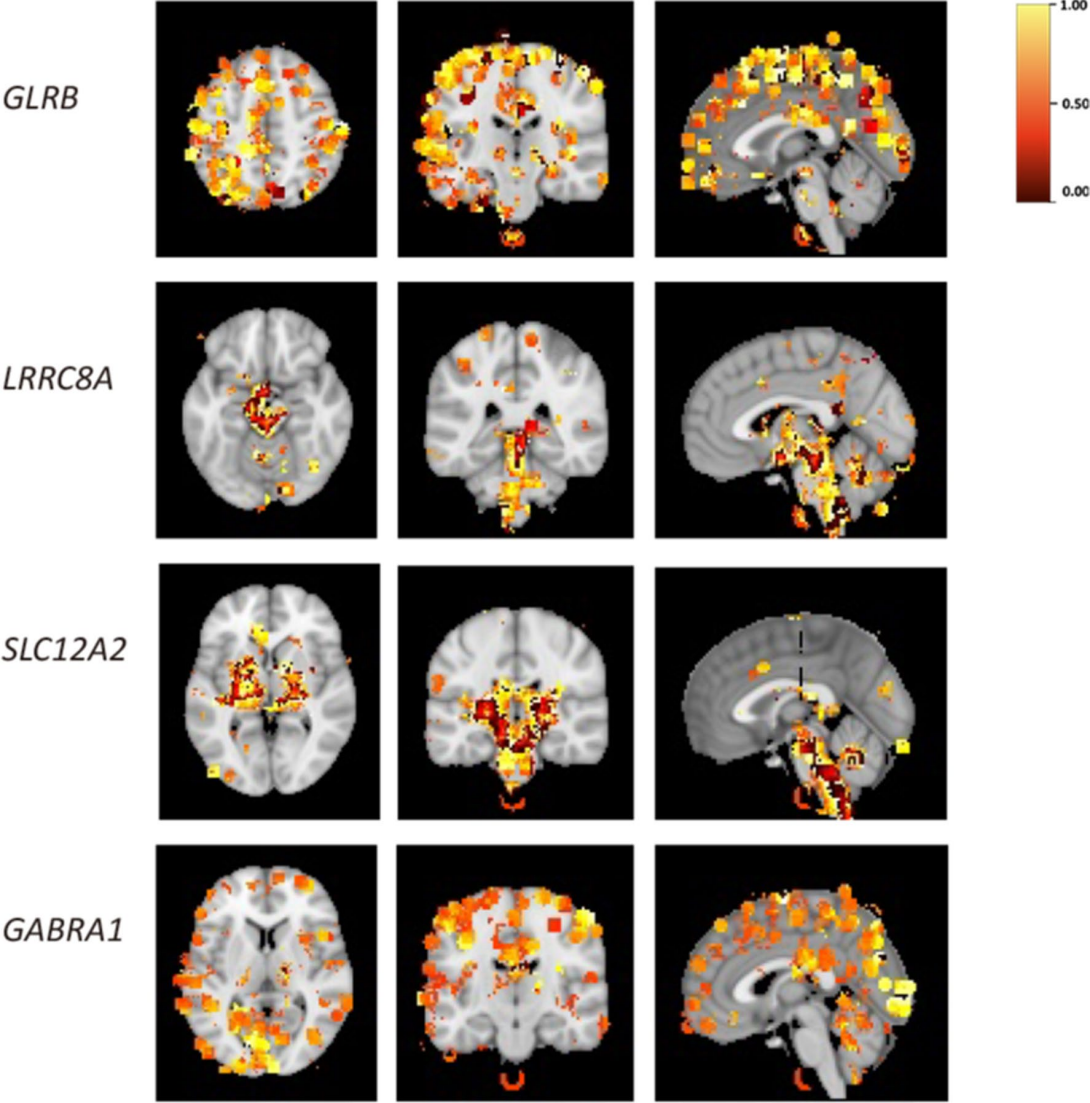
Spatial expression patterns of GClTC in the brain. These figures show the brain regions with high levels of GClTC. Representative genes were selected from different clusters. The heatmap indicates a correlation; yellow indicates a relatively high correlation, while red indicates a relatively low correlation. The thresholds followed the settings we have set in Neurosynth.org. *GLRB* in cluster 4, is mainly expressed in the cerebral cortex. *LRRC8A* in cluster 6, is mainly expressed in the brainstem, midbrain, and thalamus. *SLC12A2* in cluster 8, is mainly expressed in the thalamus, brainstem, and basal ganglia. *GABRA1* in cluster 9, is predominantly expressed in the cerebral cortex, particularly in the visual cortex and parietal lobe

To explore the spatial expression features of GClTC clusters in the brain, we downloaded spatial gene expression maps of specific genes of interest from the Neurosynth [23]. Neurosynth is a large database of mappings structured by text-mining, meta-analysis, and machine-learning techniques. We used the Neurosynth-Gene module to explore gene expression levels in a whole-brain map. The distribution of all genes of interest is shown in Additional file 10: Fig. S3 and Additional file 5: Table S5, and the representative genes from different clusters are shown in Fig. 5. We found that the members of cluster 4 were observed with relatively higher expression levels in the cortical area. The GClTC in cluster 6 were mainly expressed in the basal brainstem and ganglia. Moreover, the GClTC in cluster 8 were also highly expressed in the brainstem, basal ganglia, and thalamus. The gene expression levels in cluster 9 were higher in the visual cortex, temporal lobe, parietal lobe, and hippocampus. These results showed that GClTC present anatomical heterogeneity in different clusters.

### GClTC enriched for different CNS disorders

**Fig. 6:**
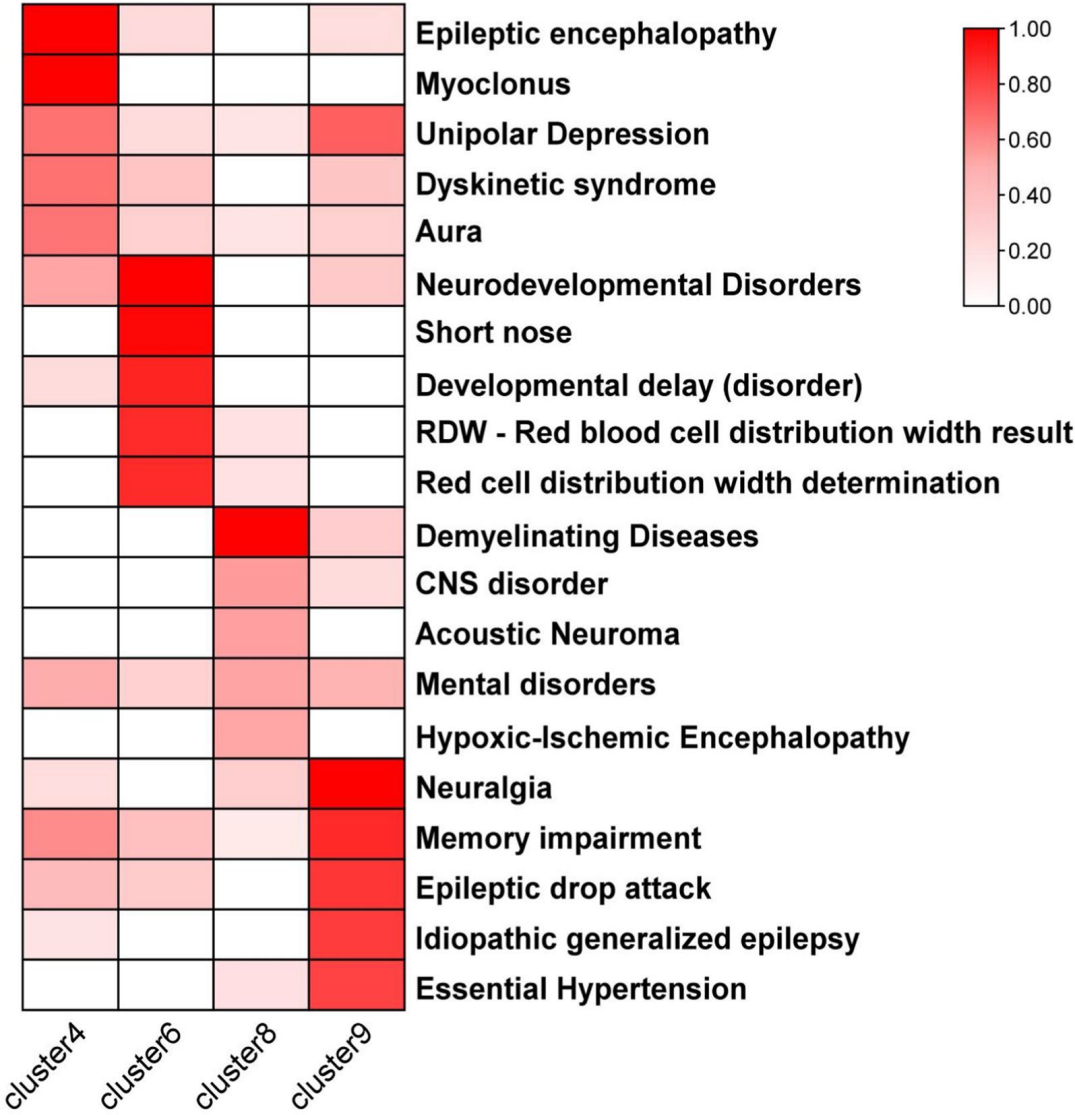
Gene-disease association analysis of genes in different clusters. The heatmap shows the zero to one transformed values of the disease terms in the gene-disease association analysis describing each of the 4, 6, 8, and 9 clusters

The GClTC from different clusters have unique expression characteristics. As the genes in clusters 4, 6, 8, and 9 were enriched for some CNS-specific terms, we further investigated the connections between those genes and diseases. The DisGeNET database, which offers rich information of genes and variants associated with many kinds of human diseases, was used to enrich for the diseases with all genes in clusters 4, 6, 8, and 9. We observed that genes from these 4 clusters were enriched for distinct diseases, most of which were CNS disorders (Fig. 6 and Additional file 6: Table S6). Clusters 4 and 9 were positively and similarly enriched for epilepsy. Cluster 6 was enriched for neurodevelopmental disorders. “Demyelinating diseases” was glaringly enriched in cluster 8.

As shown in our findings (Figs. 3, 4, 5), GClTC in different clusters exhibited functional and anatomical heterogeneity. We speculated that the heterogeneity might account for their different roles in different CNS diseases. To test our hypothesis, we selected typical GClTC to illustrate the intrinsic connection between their expression features and CNS diseases.

**Fig. 7:**
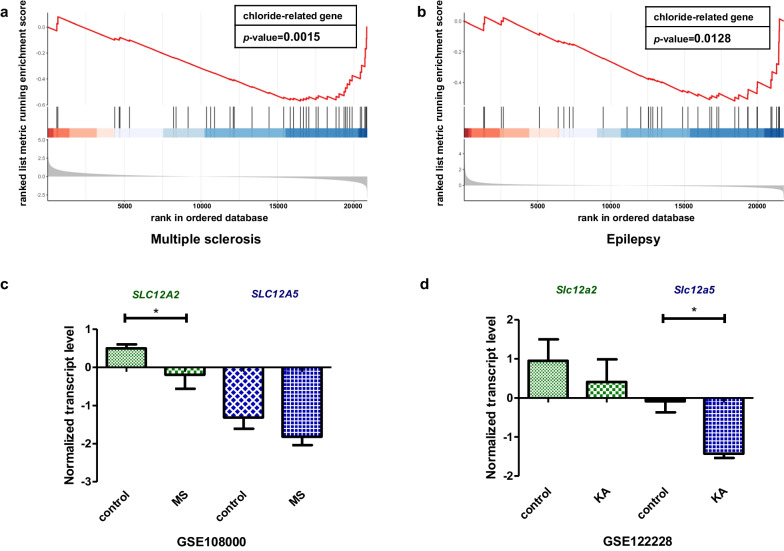
*SLC12A2* and *SLC12A5* mRNA levels in different CNS disorders. **a** The GClTC set enrichment analysis between health control and MS patient (*p* = 0.002). **b** The GClTC enrichment analysis between control and KA (Kainic acid)—induced epileptic model (*p* = 0.013). **c** *SLC12A2* mRNA (*W* = 54, *p* = 0.035, Wilcoxon test), *SLC12A5* mRNA (*W* = 43, *p* = 0.237, Wilcoxon test) normalized expression level in health control and MS patient. Values represent the median (IQR). **d** *Slc12a2* mRNA (*t*(4)  = 0.683, *p* = 0.532, Student's t-test) and *Slc12a5* mRNA (*t*(4) = 4.446, *p* = 0.028, Student's t-test) normalized expression level in health control and epileptic model. Values represent mean ± SD. *p < *0.05*

The neuronal chloride ion gradient is primarily established by cation-chloride cotransporters (CCCs) from the SLC12A gene family, especially the chloride ion importer sodium potassium chloride cotransporter-1 (NKCC1) and the chloride ion exporter potassium chloride cotransporter-2 (KCC2). NKCC1 and KCC2 encoded by *SLC12A2* and *SLC12A5*, respectively [31]. The abnormal transcriptional regulations of these two genes lead to multiple neurological disorders resulting from disrupted chloride homeostasis. According to the previous analysis (Table 1 and Fig. 6), *SLC12A2* was classified in cluster 8 related to demyelinating diseases, while *SLC12A5* was classified in cluster 9 related to epilepsy. To further confirm their relationship with the enriched diseases, we examined their heterogeneity in two Gene Expression Omnibus (GEO) datasets, multiple sclerosis (MS) (GSE108000) (one of the most common demyelinating diseases) and epilepsy (GSE122228), both of which were strongly associated with chloride homeostasis disorder [32, 33]. We next defined 104 GClTC as a gene set (Additional file 2: Table S2). GSEA was conducted to determine whether those genes showed statistically significant, concordant differences in MS and epilepsy. The results showed that the defined gene set was significantly enriched in multiple sclerosis (*p* < 0.05) (Fig. 7a) and epilepsy (*p* < 0.05) (Fig. 7b), indicating that GClTC were involved in disease mechanisms. Then, we compared mRNA levels of *SLC12A2* and *SLC12A5* in MS and epilepsy separately (Fig. 7c, d and Additional file 7: Table S7). *SLC12A2* showed no significant difference in models with epilepsy compared to the controls (control mean ± SD = 0.952 ± 0.943, KA mean ± SD = 0.412 ± 0.994, *p* = 0.532, Student's t-test), but it was significantly decreased in multiple sclerosis [control median (IQR) = 0.511 (0.392), MS median (IQR) = 0.200 (0.597), *p* = 0.035, Wilcoxon test]. In contrast, *SLC12A5* expression levels were significantly decreased in epilepsy (control mean ± SD = − 0.086 ± 0.486, KA mean ± SD = − 1.428 ± 0.192, *p* = 0.028, Student's t-test) but not in demyelinating diseases [control median (IQR) = − 1.530 (1.246), MS median (IQR) = − 1.955 (0.451), *p* = 0.237, Wilcoxon test]. This result implied that the expressions of *SLC12A5* and *SLC12A2* had different effects on the pathological mechanisms of these two diseases.

## Discussion

Abnormal chloride homeostasis occurs in various CNS disorders; however, the underlying mechanisms are not yet fully understood. Chloride homeostasis is delicately regulated by various GClTC, so we explored the relationship between GClTC and neurological diseases according to their temporal and spatial expression patterns and provided a new perspective to illustrate the intrinsic connections between them.

As temporal demands drive the evolution of neural diversity, time is regarded as a key metric for all brain operations. With neural signals sending out specific programs to start or stop neurogenesis, distinct functions are generated in specific time windows during neuronal development, which is driven by multiple molecular events [34, 35]. Therefore, those gene expression that change synchronously during development may play a key role in the establishment of brain functions from simple to complex. Thus, understanding the transcriptional dynamics of crucial genes may help interpret the functional heterogeneity underlying neurodevelopmental processes. Time series analysis can help to understand related information surrounding biological processes, and it has been used in studies of a variety of species, including Drosophila, Arabidopsis thaliana, and so on [36, 37]. The STEM, a time series analysis software, allows us to screen genes with similar variation expression dynamics in mRNA levels. In this study, we used STEM to cluster genes whose expression changed synchronously during brain development. As chloride-mediated signals are involved in the formation of neural function during early development, we believe that taking a developmental perspective in investigation can help us better understand the neural regulatory mechanisms where GClTC are involved.

To illustrate the relevance between expression trends and biological functions, we clustered genes with similar expression dynamics and enriched their biological functions. Some clusters targeted enrichment terms for transcriptional regulation, division regulation, and energy metabolism, which belong to general cell functions (Additional file 8: Fig. S1). Interestingly, some enrichment terms were related to specific neural cell functions. For instance, genes in cluster 4 were enriched for the function of synaptic components, signal transmissions, and targeted neuron-related pathways such as the phosphatidylinositol signaling pathway. The phosphatidylinositol signaling system regulates cellular functions such as receptor signaling, secretion, and endocytosis. The disorder of phosphoinositide system leads to brain fail to react appropriate responsive to stimuli, which is associated with several neurological diseases [38, 39]. Therefore, we suggest that classifying genes based on their temporal expression may help screen for biological characteristics related to neurodevelopmental processes.

We further focused on the GClTC in these CNS-related clusters. Most GClTC have been reported to be involved in the corresponding functions, as shown in our results. These gene functions are consistent with the enriched term in cluster 4 (regulating synaptic signaling), Anoctamin 3 (*ANO3*) has been reported to control the excitability of synapses in the hippocampus in response to hyperthermia [40, 41]. Solute carrier family 12 member 6 (*SLC12A6)* and solute carrier family 12 member 6 (*SLC12A7*) help maintain cell volume and intracellular chloride levels [42], concordant with their functions in cluster 6. The gene tweety family member 2 (*TTYH2*) in cluster 8 constitutes the volume-regulated anion channel (VRAC) that decreases intracellular volumes during cell swelling [43, 44]; it executes glial-related functions. This suggested that GClTC from different clusters might play different and specific roles as opposed to all the GClTC playing the same role during development.

The similarities between gene in expression patterns may reflect cell type-specific expression [45]. Thus, we suppose that this distinction of functions might result from differences in the cell types. As mentioned before, clusters 4, 6, and 9 were enriched for neural functions, consistent with our finding that the GClTC in those clusters were mainly detected in neurons. For example, the family of GABA receptor genes, which are mainly expressed in neurons [46], are found in cluster 9. Furthermore, the expression of *GABRA5* and *GABRD* were mostly detected in excitatory neurons, indicating their important role in this cell type. It has been shown that *GABRA5* contributes to excitatory synapse development [47, 48]. Moreover, *GABRD* codes for the delta subunit of the GABA receptor, which generates tonic inhibition [49, 50]; its effect on excitatory neurons warrants further study. In addition, the data from STAB (Additional file 9: Fig. S2) indicated that dramatic changes in gene expression levels are present in specific cells during development, which confirm the important role of GClTC in neurological function formation. However, it should be noted that the data from STAB only reported gene expression levels at a few time points after birth; therefore, detailed studies for gene expression levels in the postnatal stage are necessary.

The brain is functionally organized into distinct regions. We speculated that GClTC with similar functions might have similar spatial distributions in the brain. Our results showed that the GClTC in the same clusters presented higher expression in specific brain areas, and the differences in distribution were shown between clusters. Considering that abnormal connections between certain brain regions appear in different diseases, we further analyzed the correspondence between gene expression distribution and diseases. We noticed that the brain regions with high gene expression levels were in accordance with the pathological sites of these diseases. For example, the genes in clusters 4 and 9 were mainly expressed in the cortex, hippocampus, and temporal lobe, which constitute seizure circuits and regulate different stages of seizures [51, 52]. The pathogenesis of epilepsy involves the disruption of synaptic transmission and membrane potential [53], which also corresponds with the functional enrichment analysis, indicating that the GClTC in clusters 4 and 9 may play more critical roles in the mechanism of seizures than other GClTC. We also noticed that some of the genes in these two clusters encode GABA receptors and that these genes were associated with a broad spectrum of epileptic syndromes. Variants in GABA receptor genes can either be associated with monogenic epileptic syndromes or constitute risk factors for genetically complex generalized epilepsies [54]. Interestingly, some genes in cluster 9 were expressed in the hippocampus, whereas genes in cluster 4 were less expressed in this region. This may provide a hint that genes in cluster 9 might have a higher therapeutic significance for epilepsy, whose pathology involves the hippocampus.

In previous studies, chloride has been identified as a signal that broadly regulates neural activity in the CNS. Thus far, we have suggested that GClTC might play specific roles in certain diseases because they have specific molecular characteristics, cell type distributions, and brain region distributions. Moreover, we suggest that disease-related chloride dyshomeostasis may not be caused by a single GClTC but rather by a group of genes with the same expression characteristics.

**Fig. 8:**
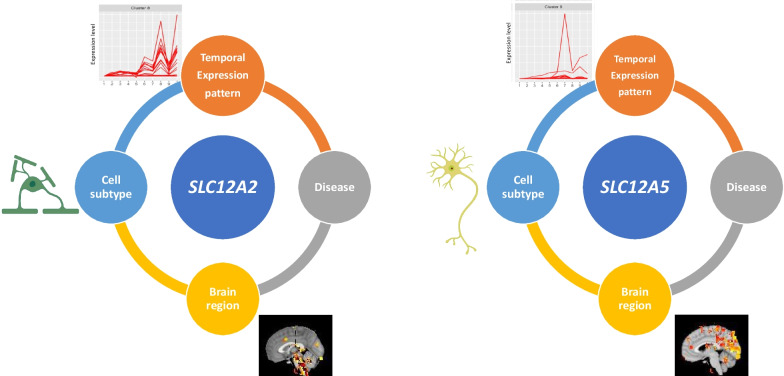
Schematic overview of the expression characteristics of *SLC12A2* and *SLC12A5. SLC12A2* in cluster 8 is enriched for the biological functions of non-neural cells, and is mainly expressed in oligodendrocytes. It is distributed in the brainstem and basal ganglia and is associated with demyelinating disease. *SLC12A5* in cluster 9 is enriched for the biological functions of neural cells. A high expression of this gene is detected in neurons. It is primarily distributed in the cerebral cortex, especially in the visual and parietal lobes, and is associated with neuralgia and epilepsy

To validate abnormal chloride homeostasis in different diseases is associated with the abnormal transcriptional levels of GClTC from different clusters, we selected two widely known GClTC, *SLC12A2* and *SLC12A5*, which encode proteins NKCC1 and KCC2, for further analysis [55, 56]. KCC2 is the major extruder of intracellular chloride, whereas NKCC1 mediates the influx of chloride ions [57]. These proteins are essential for neural chloride homeostasis. Dysfunction of them could result in the pathogenesis of several brain disorders [58] or abnormal GABAergic signal during brain development [59]. As reported in some research, *SLC12A2* presents a relatively high expression level in early development stage in brain and decreases after birth [60]. Interestingly, in temporal single-cell results (Additional file 9: Fig. S2), we found that *SLC12A2* expression remained at a low level in neurons, and the slight upregulation was only observed mainly in oligodendrocytes after birth but was stable in neurons (Additional file 9: Fig. S2). Recent research also revealed that NKCC1 protein and mRNA are expressed at remarkably high levels in oligodendrocytes in mice [61]. If dramatic changes in mRNA occur mainly in non-neuronal cells, how it regulates neuronal chloride homeostasis during development warrants further study. Moreover, based on our data, *SLC12A2* and *SLC12A5* had completely different expression patterns (Fig. 8). *SLC12A5* has a pattern similar to that of GABA receptors; it is located in neurons and is mainly involved in regulating neural functions, consistent with previous reports [62–64]. In contrast, *SLC12A2* mediates the function of non-neuronal cells. It is associated with myelin dysfunction, which has rarely been reported. Although, *SLC12A2* is regarded as a target gene in epilepsy treatment [65, 66], we found no significant changes in *SLC12A2* expression in epileptic models, but they seemed to act more sensitively in demyelinating diseases. Overall, we suggest *SLC12A2* may play a more important role in oligodendrocytes than in neurons, and myelination disorders might be much more widespread than what has been thought so far.

In summary, although chloride homeostasis disorders occur in various neurological diseases, the GClTC are specific to certain types of disease. The findings of our current study are compelling, but they have some limitations. Not all the genes relevant to chloride homeostasis were included in our gene clusters. We only focused on the chloride transporters and channels, considering they are the direct executors to maintain intracellular chloride homeostasis. However, as reported by other study [8], genes like Na–K ATPase α2 (*ATP1A2*) and synaptophysin (*SYP*) also manifested similar expression pattern as *SLC12A5*, even though these genes are not chloride-regulatory genes. The lack of abundant postnatal data on critical periods (CPs) limits the interpretation of the results in those periods. It should not be ignored that the delicate regulation of post-translational modification during development can magnify the effects of protein functions even when the mRNA expression level is relatively low. Besides, even though we observed strong association between GClTC and diseases, it is still unclear whether the changes of gene expression are the cause of these diseases, or the changes are merely the adaptive responses due to the diseases. Therefore, we believe that more comprehensive studies on GClTC are required to elucidate the intricate mechanisms underlying brain diseases.

## Supplementary Information

**Additional file 1: Table S1.** Definitions of the periods of human development.

**Additional file 2: Table S2.** The time series analysis of genes in cluster 1 ~ 10.

**Additional file 3: Table S3.** List of 104 GClTC.

**Additional file 4: Table S4.** The top 5 significant terms enriched from cluster 1 ~ 10.

**Additional file 5: Table S5.** Distribution of GClTC in brain regions.

**Additional file 6: Table S6.** List of clusters 4, 6, 8, and 9 annotated through gene-disease association analysis.

**Additional file 7: Table S7.** The normality and variance homogeneity for data in Fig. 7.

**Additional file 8: Fig. S1.** Molecular characteristics of the developmental expression patterns for 1–10 cluster.

**Additional file 9: Fig. S2.** Gene expression dynamics across cell subtypes of the selected brain region.

**Additional file 10: Fig. S3.** Distribution of neural GClTC in different brain regions.

## Data Availability

Data will be made available on reasonable request.

## Abbreviations

*CNS*: Central nervous system
*ASD*: Autism spectrum disorder
*STAB*: Spatio-temporal cell atlas of the human brain
*MS*: Multiple sclerosis
*KA*: Kainic acid
*IQR*: Interquartile range
*CPs*: Critical periods
