# Supplementary material for: Spatiotemporal expression patterns of genes coding for plasmalemmal chloride transporters and channels in neurological diseases
Source: Mol Brain. 2023 Mar 18;16:30. doi: 10.1186/s13041-023-01018-w (PMC10024392; doi:10.1186/s13041-023-01018-w)
Supplement: Supplementary file 1 — Additional file 1: Table S1. Definitions of the periods of human development. [file 13041_2023_1018_MOESM1_ESM.pdf]

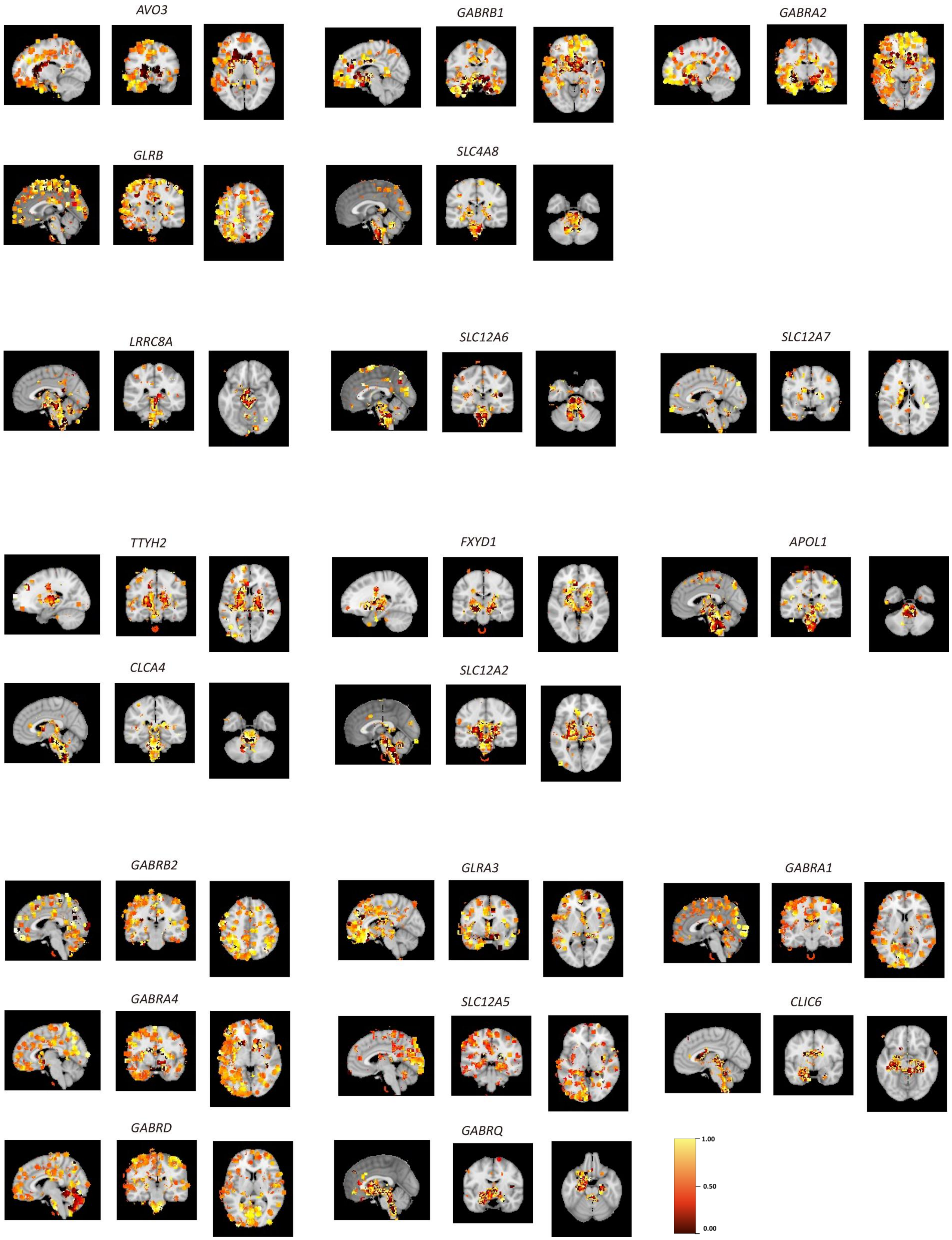

Fig.S3 Distribution of chloride-related genes in different brain regions. These figures show the brain regions with high levels of chloride-related genes. The heat map indicates a correlation; yellow indicates a relatively high correlation, while red indicates a relatively low correlation.
