# Supplementary figures and images for: Spatiotemporal expression patterns of genes coding for plasmalemmal chloride transporters and channels in neurological diseases
Source: Mol Brain. 2023 Mar 18;16:30. doi: 10.1186/s13041-023-01018-w (PMC10024392; doi:10.1186/s13041-023-01018-w)

a

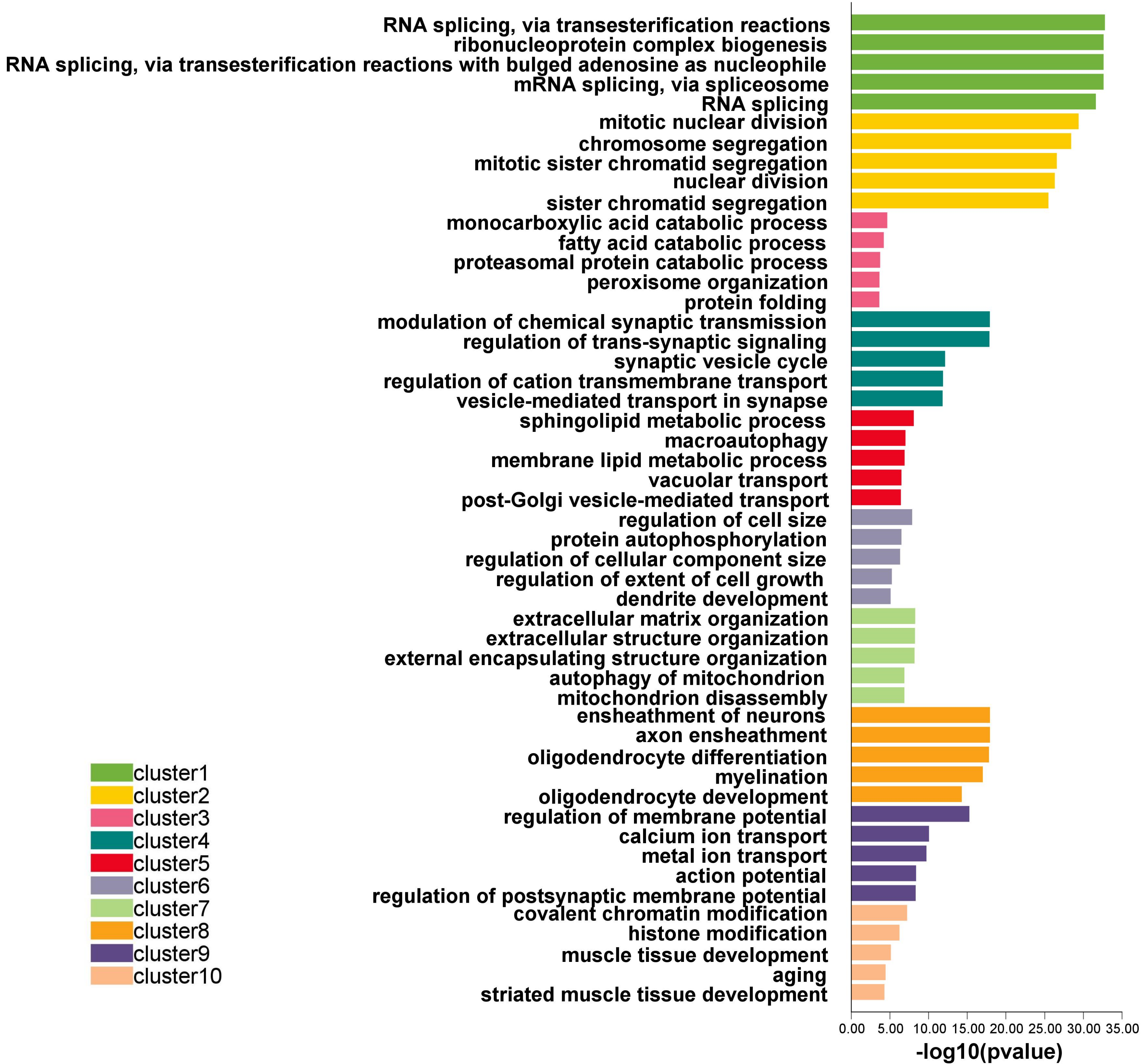

b

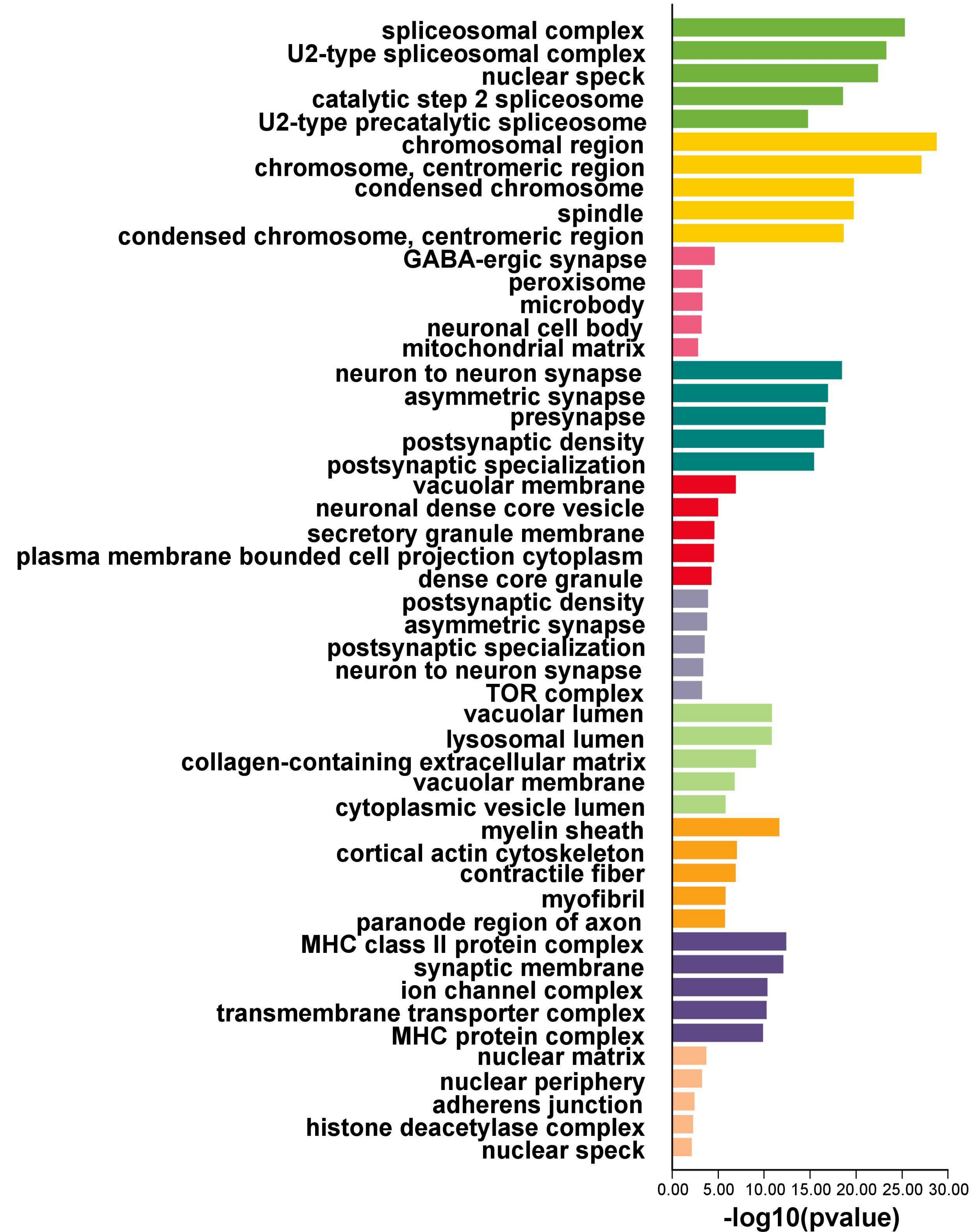

c

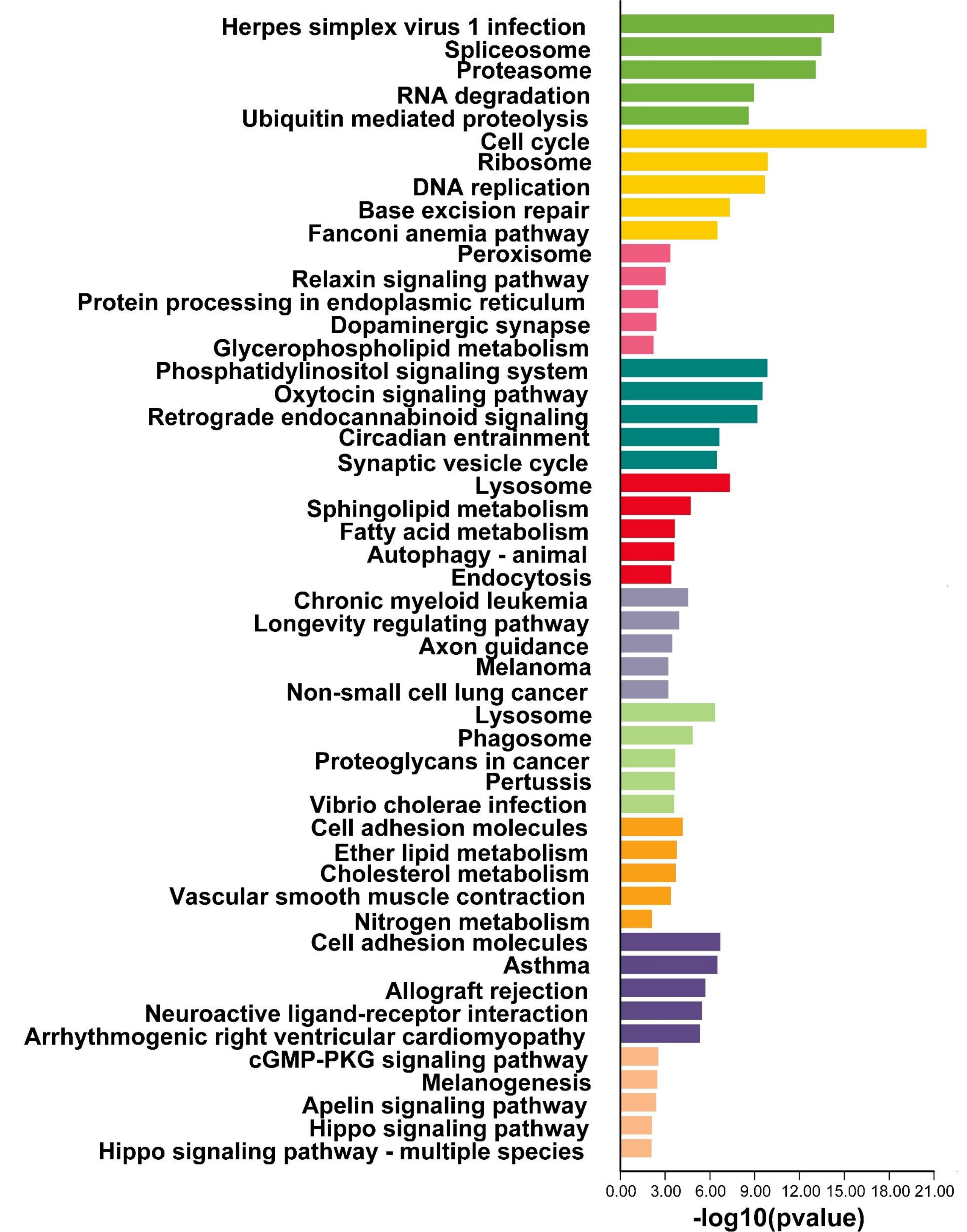

Supplement: Supplementary file 9 — Additional file 9: Fig. S2. Gene expression dynamics across cell subtypes of the selected brain region. [file 13041_2023_1018_MOESM9_ESM.pdf]
