# Supplementary material for: Spatiotemporal expression patterns of genes coding for plasmalemmal chloride transporters and channels in neurological diseases
Source: Mol Brain. 2023 Mar 18;16:30. doi: 10.1186/s13041-023-01018-w (PMC10024392; doi:10.1186/s13041-023-01018-w)
Supplement: Supplementary file 10 — Additional file 10: Fig. S3. Distribution of neural GClTC in different brain regions. [file 13041_2023_1018_MOESM10_ESM.pdf]

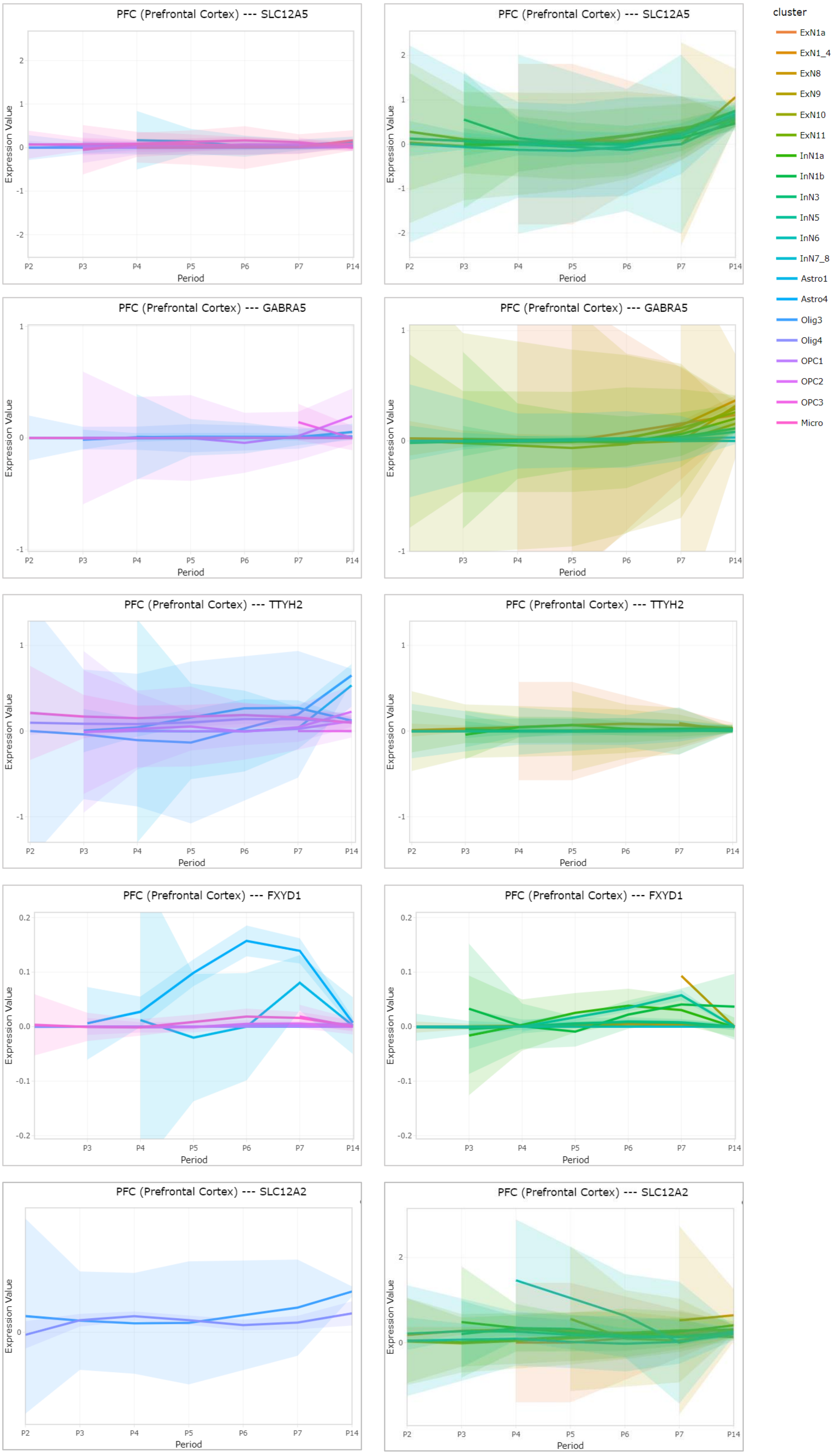

Fig.S2 Gene expression dynamics across cell subtypes of the selected brain region. The figures on the left show the gene expression changes in non-neuronal cells. The figures on the right show the gene expression changes in neuronal cells. P2: 8<= age < 10 pcw; P3: 10<= age < 13 pcw; P4: 13<= age < 16 pcw; P5: 16<= age < 19 pcw; P6: 19<= age < 24 pcw; P7: 24<= age < 38 pcw; P14: 40<= age < 60 yrs. ExN, excitatory neuron; InN, inhibitory neuron; Astro, astrocyte; Oligo, oligodendrocyte; OPC, oligodendrocyte Precursor Cell; Micro, microglial cell.
